# Resection of an anterolateral mesencephalic cavernoma via transsylvian/transuncal approach in a child

**DOI:** 10.3171/2019.7.FocusVid.19149

**Published:** 2019-07-01

**Authors:** Sahin Hanalioglu, Omer Selcuk Sahin, Mehmet Erhan Turkoglu

**Affiliations:** Department of Neurosurgery, Health Sciences University, Diskapi Yildirim Beyazit Training and Research Hospital, Ankara, Turkey

**Keywords:** cavernous malformation, brainstem, midbrain, transsylvian, transuncal, video

## Abstract

This video demonstrates the resection of an anterolateral mesencephalic cavernous malformation (CM) through a transsylvian/transuncal approach. A 10-year-old girl presented with progressive headache and left-sided spastic hemiparesis. Neuroimaging revealed a 20-mm CM located in the right anterolateral midbrain/cerebral peduncle. After orbitozygomatic craniotomy and wide sylvian fissure opening, the oculomotor nerve was dissected and separated from the temporal lobe. Partial resection of the uncus allowed access to the CM through the oculomotor-tentorial triangle. The CM was excised in a piecemeal fashion. Postoperative imaging confirmed the gross-total resection. The patient had no additional neurological deficits postoperatively. Her left hemiparesis almost completely resolved at the 12-month follow-up.

The video can be found here: https://youtu.be/Jb_EaWbn5LU.

## Transcript

In this 2D operative video, we present a pediatric patient with a midbrain cavernous malformation who underwent microsurgical resection via transsylvian/transuncal approach.

### 0:32 Clinical presentation

The patient is 10-year-old female who presented with 2-month history of worsening headache and left-sided spastic hemiparesis. Her medical history revealed that 6 months ago, she had a similar attack with headache and mild left hemiparesis which resolved spontaneously within a few days. The patient was referred to us for neurosurgical evaluation.

### 0:54 Preoperative imaging

MRI shows a 2-cm cavernous malformation located in right anterolateral midbrain causing displacement of corticospinal tracts medially. The lesion appears to expand the cerebral peduncle and reach the anterolateral surface of mesencephalon. It causes obliteration of the crural and ambient cisterns, and compression of vascular structures within, such as posterior cerebral artery and basal vein of Rosenthal, and also medial temporal structures (Szabo et al., 2017).

### 1:22 Surgical alternatives

Although subtemporal approach with or without transtentorial extension, paramedian or extreme lateral supracerebellar infratentorial approach, or an orbitozygomatic craniotomy with a combination of transsylvian and pretemporal approaches can all be considered suitable surgical alternatives, we decided to perform a transsylvian/transuncal approach taking into account the imaging features of the lesion and individual anatomy (Bilginer et al., 2014; Cavalcanti et al., 2018; Delaunois et al., 2018; Kalani et al., 2016; Mascitelli et al., 2019a; Munich and Morcos, 2019).

### 1:47 Surgical strategy

Therefore, under intraoperative neurophysiological monitoring, the patient underwent microsurgical gross-total resection of the cavernoma via an orbitozygomatic craniotomy followed by transsylvian/transuncal approach. Oculomotor-tentorial triangle was used to maximize the surface area accessed through this approach (Kalani et al., 2016; Seçkin et al., 2008; Mascitelli et al., 2019b).

### 2:09 Opening

The patient was placed supine and the head was slightly rotated toward left side. A wide pterional skin incision was made from in front of the tragus to the midline coursing behind the hairline. After dissection and retraction of temporal muscle, a relatively large orbitozygomatic craniotomy was performed.

Dura was opened in C-shape fashion and reflected anteriorly towards the orbit and lateral sphenoid wing.

### 2:33 Microscopic stage: sylvian fissure dissection

In addition to brain relaxation with mannitol administered during the craniotomy, right after the dural opening, CSF is drained while the frontal lobe is retracted gently. Arachnoidal dissection is performed to split sylvian fissure as wide as possible. We first started dissection from proximal sylvian fissure to effectively drain CSF and relax the brain in the beginning of the surgery. But it can be done from distal to proximal as well. Arachnoidal adhesions are dissected and divided with microscissors carefully. Care is taken not to damage MCA branches and bridging veins.

At this stage, we move to more distal portion of sylvian fissure and carefully dissect M2 and M3 branches from adjacent temporal and frontal lobes. Mild retraction of temporal lobe helps to release adhesions; however, forceful retraction should be avoided.

All major branches of the right ICA and MCA can be seen at the moment. Dense arachnoid membrane covering opticocarotid triangle is dissected and cut to access carotid cistern.

### 3:44 Microscopic stage: oculomotor-tentorial triangle

Through the carotico-oculomotor triangle, the membrane of Liliquist is visualized and carefully inspected before cutting in order to avoid possible damage to perforators. The oculomotor nerve is circumferentially dissected and freed from adjacent arachnoids throughout the cistern down to the brainstem. The nerve is carefully separated from the posterior cerebral artery. The oculomotor-tentorial triangle is now exposed.

Thick arachnoidal folds tethering uncus are dissected and divided to widen the exposure. Maximal care is exercised to preserve posterior communicating and anterior choroidal arteries and their perforators. Tiny perforators can easily be confused with arachnoid bands; therefore, larger magnification and careful inspection may be helpful. Further dissection and lateral retraction of temporal lobe allows partial visualization of the cavernoma.

### 4:48 Microscopic stage: partial resection of uncus

Attempts are made to broaden the exposure of the lesion but are not successful due to the large size of the cavernoma. This limited exposure appears to be insufficient, and also further retraction is not desired to avoid venous infarction. So, we decide to perform partial resection of uncus to expose surface of cavernoma more laterally. Now posterior cerebral artery is better visualized. Bipolar coagulation and suction are used to partially remove the uncus until we see the most lateral aspect of the lesion. Since vasospasm is suspected at this stage, micro-Doppler is used to check its flow patency before starting the resection of cavernoma.

### 5:44 Microscopic stage: resection of the lesion

Midbrain is incised in the area where the lesion comes closest to the surface and away from the corticospinal tracts. Some thrombosed parts can be well circumscribed and separated from the brainstem. On the contrary, subacute and chronic hemorrhagic areas can be difficult to dissect from surrounding hemosiderin-stained brainstem. Thus, piecemeal resection is preferred to minimize damage to critical nuclei and white matter tracts. Under neurophysiological monitoring, resection of the cavernous malformation is carried out with gentle dissection and traction with the use of forceps and suction. Small vessels connecting the cavernous malformation and surrounding tissues can be coagulated and divided. We continue piecemeal resection until complete excision. After the resection, surgical cavity is inspected for residual cavernoma. Finally, meticulous hemostasis is performed.

### 6:40 Closure and postoperative imaging

Standard closure is carried out with watertight dural closure and replacement of bone flap with plates. Postoperative MRI shows complete resection of the cavernous malformation. Tractography images show largely conserved corticospinal fibers.

Postoperatively, the patient had no additional neurological deficits. And late follow-up exams showed markedly improved left hemiparesis.
